# The Effects of Physical Exercise on Mental Health: From Cognitive Improvements to Risk of Addiction

**DOI:** 10.3390/ijerph182413384

**Published:** 2021-12-19

**Authors:** Pasquale Caponnetto, Mirko Casu, Miriam Amato, Dario Cocuzza, Valeria Galofaro, Alessandra La Morella, Sara Paladino, Kamil Pulino, Nicoletta Raia, Flavia Recupero, Cristian Resina, Samuele Russo, Laura Maria Terranova, Jennifer Tiralongo, Maria Chiara Vella

**Affiliations:** 1 Department of Educational Sciences, University of Catania, 95123 Catania, Italy; miriam.amato1998@gmail.com (M.A.); dariococuzza01@gmail.com (D.C.); galofaro.valeria@tiscali.it (V.G.); alessandralamorella@gmail.com (A.L.M.); sara_paladino@hotmail.com (S.P.); kamilpulino17@gmail.com (K.P.); Nikoxx22@gmail.com (N.R.); flarecupero92@gmail.com (F.R.); cristian97ct@gmail.com (C.R.); SamRush190398@gmail.com (S.R.); Terranova-laura@virgilio.it (L.M.T.); jennitiralongo@icloud.com (J.T.); chiaravella15@gmail.com (M.C.V.); 2 Department of Clinical and Experimental Medicine, Center of Excellence for the Acceleration of Harm Reduction (CoEHAR), University of Catania, 95123 Catania, Italy; 3 Center for Tobacco Prevention and Treatment, University Hospital “Policlinico G.Rodolico-San Marco”, University of Catania, 95123 Catania, Italy

**Keywords:** physical activity, exercise addiction, sport addiction, cognitive functions, executive functions

## Abstract

(1) *Background*: we aimed to investigate the effects of physical activity on cognitive functions and deficits of healthy population and other needy groups. Secondly, we investigated the relation between healthy habits and psychopathological risks. Finally, we investigated the impact of COVID-19 pandemic on exercise addiction and possible associated disorders. (2) *Methods*: From April 2021 to October 2021, we conducted a review aimed at identifying the effects of physical exercise on mental health, from cognitive improvements to risk of addiction; we searched for relevant studies on PubMed, Web of Science, EMBASE, PsycINFO and CINHAL. (3) *Results*: For the first purpose, results indicated multiple effects such as better precision and response speed in information processing tasks on healthy populations; improvement of executive functions, cognitive flexibility and school performance in children; improvement of attention and executive functions and less hyperactivity and impulsiveness on children with attention deficit hyperactivity disorder (ADHD); improvement of executive and global functions on adults; improvement of overall cognitive functioning on patients with schizophrenic spectrum disorder or bipolar disorder. Data also demonstrated that exercise addiction seems to be related to low levels of education, low self-esteem, eating disorders and body dysmorphisms. Eventually, it was found that people with lower traits and intolerance of uncertainty show a strong association between COVID-19 anxiety and compulsive exercise and eating disorder. (4) *Conclusions*: these findings underline on one side the beneficial effects of physical activity on cognitive function in healthy individuals in a preventive and curative key, while on the other side the importance of an adequate evaluation of psychological distress and personality characteristics associated with exercise addiction.

## 1. Introduction

According to the World Health Organization (WHO) definition, physical exercise is “any body movement made by skeletal muscles which requires waste of energy”: this last one includes whatever everyday physical activity, and it is considered a determinant of a healthy lifestyle. Differently, “sport training” represents a lower-ranking classification compared to physical exercise, characterized by planning, organization, temporal repetitiveness and a final or intermediate aim to improve, or maintain, one or many physical well-being factors. Physical exercise is an important element which leads to beneficial effects in terms of physical and cognitive human being functioning [1].

“Cognitive processes” are defined as specific mental functions such as: memory (conceived in its different forms), attention (both general and selective), language, praxis functions (which is the capacity of making finalized movements to reach a result) and gnostic functions (namely being able to perceive and recognize). Lastly, there are the so-called “executive functions”: the ability to plan, control and coordinate thoughts and actions [2].

Health professionals should promote sport as an element of cognitive stimulation but at the same time also identify those rare cases in which a sport addiction can occur.

The aim of this review is to investigate the relationship between physical activity and cognitive functions by putting the stress on both positive effects and negative ones too (if there are any); such an objective was achieved.

There are several beneficial effects of physical activity: for instance, exercise helps to avoid physical and mental issues in children and adults by promoting neuronal plasticity, [3], making the cognitive decline in the elderly slower and improving deficient conditions in the youth who suffer from development disorders [4]. Moreover, another positive aspect may well be the strengthening of social and relational skills [5]. Furthermore, physical exercise can be considered as a possible form of therapy both in the treatment of psychopathologies and neurodegenerative illnesses [6] and as an alternative and complementary therapy too [4]. Recent studies have shown the importance of physical activity thanks to its capacity of protecting the immune system during the COVID-19 pandemic [7]. In addition, physical exercise can attenuate anxiety, stress, and depression symptoms, improving sleep disorders [8]. Nevertheless, anxiety and depression can be associated to compulsive physical exercise [9] since there is a weak boundary between sport positive aspects and the risk of determining dependence: the actual research, indeed, also underlines the cons of doing sport (for instance, the exercise addiction might cause relevant psychophysical consequences) [10].

Exercise addiction is a psychological disorder characterized by a compulsive engagement in any form of physical exercise, despite negative consequences. While regular exercise is generally a healthy activity, exercise addiction generally involves performing excessive amounts of exercise to the detriment of physical health, spending too much time exercising to the detriment of personal and professional life, and exercising regardless of physical injury [11,12,13]. It may also involve a state of dependence upon regular exercise which involves the occurrence of severe withdrawal symptoms when the individual is unable to exercise [11]. In fact, Aidman and Woolard suggested that the symptoms that appear in athletes 24–36 h after missing a planned training session when it is impossible or canceled should be used as the criteria for the diagnosis of exercise addiction. These withdrawal signs include anxiety; restlessness; a feeling of guilt, tension, and discomfort; apathy; sluggishness; lack of appetite; sleeplessness; and headaches [14]. Exercise addiction also shows a high comorbidity with eating disorders [12].

This type of addiction can be classified under a behavioral addiction in which a person’s behavior becomes obsessive, compulsive, and/or causes dysfunction in a person’s life [14]. Among DSM-5 criteria for exercise addiction there are: an increase in tolerance, withdrawal symptoms, loss of control, doing physical exercise for too much time, reduction of other activities, continuation of physical activity even if there are physical, psychological, and relational problems [15]. Concerning scientific literature, previous studies highlighted correlations between personal traits and personality [16], and behavioral disorders linked to exercise addiction [17], also analyzing both genetic and environmental causes [18].

Other meta-analyses and systematic reviews focused on the cognitive benefits of physical activity and other meta-analyses and systematic reviews focused on the physical activity addiction. To the best of our knowledge, no reviews were conducted specifically on the effects of physical exercise on mental health and explore the direction from cognitive improvements to risk of addiction. Our aim is to focus specifically on effects of physical exercise providing an updated view of the current spectrum of knowledges about sport, health benefits and potential risks.

This review led to delineate different aspects of the relationship between sport and cognitive function. On the one hand, its benefits, on the other, the risks that can arise when sports practice leads to addiction.

In addition, the ongoing COVID-19 pandemic has changed many aspects of daily life; during the first global lockdown in March 2020, numerous people were forced to stay at home to prevent further contagion of the virus. Research studies were carried out on physical activity during that period [19], with particular focus on implementing it to provide immune protection against long-term SARS-CoV-2 virus infection [20,21] and to avoid excessive sedentary lifestyle [19]. With the imposition of staying at home and the consequent stress of the global situation, an increased use of physical activity and physical exercise as a form of psychophysical release has been hypothesized, especially to combat boredom and reduce stress [22]; this could have led to the development of compulsive use of physical exercise, also resulting in the development of exercise addiction [15]. We have, therefore, dealt with comparing the few studies in the literature that have investigated the impact of the pandemic on exercise addiction and possible associated disorders.

From now on such topics will be examined in depth in the attempt of providing a clear and schematic recap of materials, methods, and results in order to discuss and find new future perspective research.

## 2. Materials and Methods

### 2.1. Search Strategies

From April 2021 until the date of submission of the article (October 2021), the reviewers A.L., C.R., D.C., F.R., J.T., K.P., L.M.T., M.A., M.C., M.C.V., N.R., S.P., S.R. and V.G. (please see section Author Contributions) searched first the databases PubMed, Web of Science, EMBASE, PsycINFO and CINHAL for relevant studies using the following search terms string: (“physical activity” OR “sport”) AND (“cognitive functions” OR “cognitive deficits”). Later the same reviewers searched all the databases for relevant studies using the following search terms strings: (“exercise addiction”) AND (“COVID-19 pandemic”). The electronic searching was supplemented by hand-searching of reference lists of the included review articles to identify any additional source. This review was fully conducted according to PRISMA guidelines 2020 for Systematic Reviews [23].

### 2.2. Eligibility Criteria

We included every article meeting the following criteria:

(a) All studies and review published on indexed journals and indexed in PubMed, Web of Science, EMBASE, PsycINFO and CINHAL.
(b) Studies related to:
  i Effects of PA and PE on cognitive functions or deficits.
  ii Exercise addiction, risks, symptomatology, and consequences, also in relation to COVID-19 pandemic.
(c) Written in English.
(d) Published from 2011 until the date of submission of the article.

## 3. Results

### 3.1. Characteristics of the Included Studies

**Figure 1:**
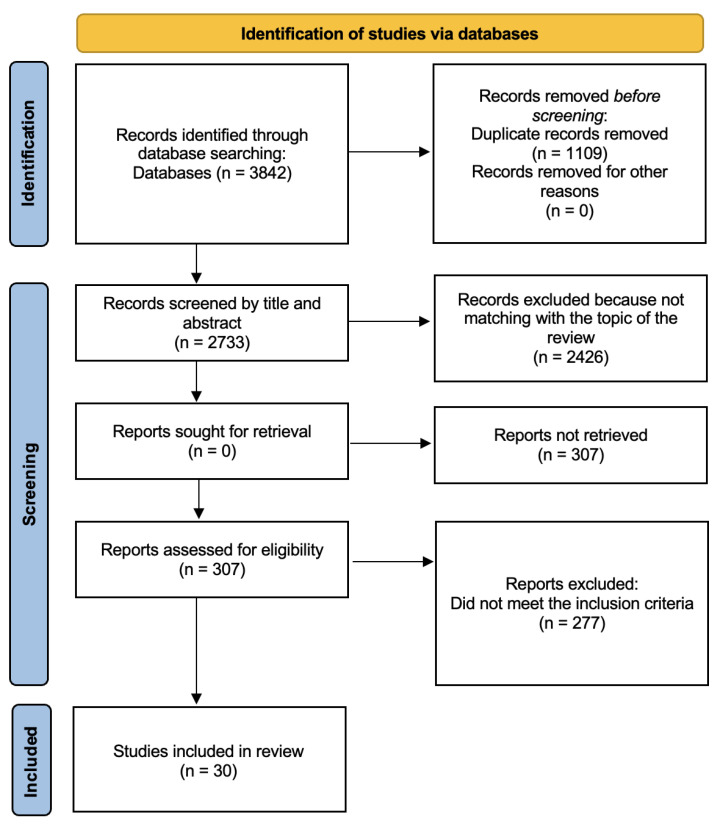
PRISMA (2020) Flow Diagram.

The database search identified a total of 3842 articles. After excluding duplicates, we found 2733 unique records, which were initially screened by reviewers S.P., J.T., S.R., A.L., M.C.V. and L.M.T. (please see section Author Contributions), based on title and abstract data. The screening has selected 307 articles to assess for eligibility criteria, 2426 records have been excluded because not matching with the intent of the present review. Three-hundred-and-seven full-text articles were assessed for eligibility, thirty out of these met the inclusion criteria and were included in the review and 277 were excluded because they did not meet the inclusion criteria (flow diagram, Figure 1).

### 3.2. Benefits of Physical Exercise on Cognitive Functions

The analysis of several research from scientific literature confirmed the positive influence of physical exercise on cognitive functions.

The phenomenon of positive influence of physical exercise on cognitive functions is evident in a randomized study conducted by Chiu et al. [24] in which the sample consisted in thirty-one participants recruited from National Central University, Taiwan. Twelve of these were in the exercise group, who regularly engaged in running or swimming, 11 were members of the university volleyball teams, and 8 were controls. For each, height, weight, and gender were recorded, and fitness was evaluated using a standard measure, the Progressive Aerobic Cardiovascular Endurance Run (PACER) test, a measure of maximal oxygen uptake (VO_2max_) and an indicator of aerobic physical fitness. A between-subjects design was used with repeated measures of flanker task accuracy and response times (for each of three different time limits for making a response) as within-subject factors. The flanker task measures information processing skill in different time constraints. Response times were used in diffusion model analysis to allow assessment of further relevant within subject parameters. Each participant performed blocks of the flanker task with a fixed response time limit (of which they were informed) for each block, with block orders (and hence the order of presentation of the time limits) being randomized across participants. Results showed that sporting participation, and more specifically, playing volleyball, was associated with better performance on the flanker task, primarily in terms of accuracy on the task but also with a trend toward faster responding. Additionally, time pressure was associated with reduced accuracy on the task. Response times also showed the expected reduction with the shorter time limits. The pattern of the effects on accuracy for the sporting groups showed more accurate performance for the volleyball group for the shortest time limit compared to controls, with seemingly intermediate performance for the exercise group. The response time data pointed out a trend toward faster responding for the volleyball group for the 1000 ms (intermediate) and 3000 ms (longest time limit) conditions.

#### 3.2.1. Effects of Physical Activity on Cognitive Functions in Relation to Age

Erickson et al. [25] also showed that physical activity (PA) improves cognitive functions in many age ranges. In their general review, they found out that both acute and chronic moderate-to-vigorous PA interventions improved brain structure and function, as well as cognition, and academic outcomes, in children from 6 to 13 years old. They refer to chronic PA behavior as PA that is repeated and lasts longer than a single session or episode. Thus, acute PA research reflects the immediate (transient) response to a single bout of PA, while chronic PA reflects a true change in an individual’s baseline (i.e., a prolonged/permanent shift in activity). In the case of chronic PA, the change is not as tightly coupled in time to the last bout of PA. Anyway, moderate evidence from randomized controlled trials (RCTs) indicated an association between moderate-to-vigorous intensity PA and improvements in cognition, including performance on academic achievement and neuropsychological tests, such as those measuring processing speed, memory, and executive function. Two systematic reviews reported by Erickson et al. have described differences in brain structure and function as a result of PA in RCTs, with additional support from cross-sectional comparisons of higher and lower fit groups of preadolescents. Briefly, findings have demonstrated differences in brain structure including greater integrity in specific white matter tracts following PA interventions. Functional brain changes resulting from PA interventions have also been noted in preadolescent children. Such studies have indicated PA intervention-induced benefits to the neuroelectric system as well as changes in functional magnetic resonance imaging (fMRI) signals. Collectively, there is moderate evidence that PA is beneficial to cognition and brain structure and function during preadolescence.

A systematic review by Bidzan-Bluma et al. [3] considered the effect of physical activity on children’s cognitive functions. The authors pointed out morphological modifications of cerebral structures and improvements of cognition abilities in children, after physical exercise, analyzing data from 58 articles. Positive influence on selective attention, development of better lexical accomplishment, effective linguistic comprehension and improved syntactical and orthographical skills were also observed. The domains in which the positive effect of physical exercise is more pronounced—in the period between childhood and preadolescence—are working memory, cognitive flexibility, and inhibition control, with consequent pursuit of objectives capability. Moreover, research suggested that physical activity positively influences verbal functions, which facilitates the learning of words in a new language, leading to richer networks of words and their meanings, and also improves spelling performance, language understanding, and the detection of syntactic errors.

Available data in literature suggest that physical exercise also positively affects cognitive domains of ADHD (attention deficit hyperactivity disorder) children [8]. Specifically, a meta-analysis conducted by Cerrillo-Urbina et al. [26] examinated five trials grouped according to the intervention program: aerobic and yoga exercise. The meta-analysis included a total of 249 children diagnosed with ADHD. Of these, 230 participated in aerobic exercises and 19 in yoga exercises. The average sample size of all groups was 31.13 subjects. The meta-analysis suggests that aerobic exercise had a moderate to large effect on core symptoms such as attention, hyperactivity and impulsivity and related symptoms such as anxiety, executive function, and social disorders in children with ADHD. Yoga exercise suggests an improvement in the core symptoms of ADHD. The main cumulative evidence indicates that short-term aerobic exercise, based on several aerobic intervention formats, seems to be effective for mitigating symptoms such as attention, hyperactivity, impulsivity, anxiety, executive function and social disorders in children with ADHD.

Regarding people over 50 years old, literature showed that acute and long-term PA improves brain structures, functions, and cognition, reducing risks connected to cognitive impairment and lowering the possibility of developing dementia [25]. In a meta-analysis of 39 randomized controlled trials conducted by Barha et al. [27], training showed to improve executive functions, episodic memory, visuo-spatial functions, words fluidity, speed processing and global cognitive functions. In specific, results suggest evidence that a larger amount of PA is associated to lower risks of cognitive decline and dementia, including Alzheimer’s disease. Another meta-analysis by Sofi et al. [28] concerning 15 prospective studies which lasted from 1 to 12 years, with a total of more than 33.000 participants, revealed that larger amounts of PA were associated to a 38% lower risk of cognitive decline. A meta-analysis conducted by Beckett et al. [29] involving 10 prospective studies—which included more than 20.000 participants—showed that larger amounts of PA were associated to a 40% lower risk of developing Alzheimer’s disease.

Furthermore, regarding the prevention of age-related cognitive decline in elderly with mild cognitive impairment (MCI), a randomized trial conducted by Bisbe et al. [30] explored cognitive effects of choreographic exercise and of a multimodal physical therapy program, with two elderly parallel groups from 65 to 85 years old with amnestic MCI, which are the subjects with highest risk of developing dementia. Participants were assigned to the choreography or physical therapy group and performed exercises twice a week, of which the duration was 60 min, for a period of 12 weeks. The 36 participants were assessed at baseline and after the 12 training weeks, through physical and neuropsychological standardized evaluations. The main result of the study was an improvement in verbal memory performance, measured with the word list learning test from the Wechsler Memory Scale—Third Edition (WMS-III). Changes in Repeatable Battery for the Assessment of Neuropsychological Status (RBANS) visual memory subtest and other cognitive scores were considered as secondary results. The comparison between groups showed the following effects: the choreography group obtained more statistically significant benefits in verbal-recognition memory than physical therapy group (*p* = 0.003). Both groups showed better performance in retarded visual recall from RBANS (choreography group: *p* = 0.022; physical therapy group: *p* = 0.030). Eventually, there have been no statistically significant worsening of any neuropsychological aspect.

#### 3.2.2. Effects of Physical Activity on Cognitive Functions in Patients with Mental Disorders

Concerning the psychiatric area, Aas et al. [6] demonstrated that physical activity leads to cognitive functions improvement in patients with severe mental disorders. This was demonstrated by dividing a sample composed of 306 participants with schizophrenia or bipolar disorder and considering two groups: patients that performed physical activity for ≥90 min a week and patients that performed physical activity for <90 min a week. Through neuropsychological evaluation, it emerged that the group which performed physical activity for ≥90 min a week had better global functioning (GAF scores; *p* < 0.001). This one group also obtained higher scores in working memory (*p* < 0.001), executive functioning (*p* < 0.001), verbal memory (*p* = 0.04) and general intellectual skills (*p* = 0.02). A multiple regression analysis was executed to investigate the relation between physical exercise (as continue variable) with cognitive function, considering age, sex, and psychiatric diagnosis. There was a positive association between physical exercise and working memory (*p* = 0.006) and executive functioning (*p* = 0.006). In addition, a significant association was observed between messenger ribonucleic acid (mRNA) of brain-derived neurotrophic factor (BDNF) levels, measured in plasma through standardized procedures, and general intellectual skills, measured by Wechsler Abbreviated Scale of Intelligence (WASI; *p* = 0.037), showing a higher mRNA of BDNF level in patients with better cognitive performance. Moreover, this study highlighted a significant association between physical exercise and mRNA of BDNF levels (*p* = 0.046).

Eventually, a meta-review by Chamberlain and Grant [16] confirmed that global cognition of subjects with schizophrenia can be improved through aerobic exercises. Particularly, a systematic review and meta-analysis by Firth et al. [31] focused on seven randomized controlled trials—which involved 292 subjects with schizophrenia—revealed that aerobic exercise improves global cognitive functioning more than control conditions, which included: only table soccer, occupational therapy and treatment as usual (*p* < 0.001).

We were able to note, therefore, how the performance of physical activity produces numerous benefits on cognitive functions on all types of analyzed samples (Table 1).

### 3.3. Risk Factors in the Development of Exercise Addiction

Exercise Addiction was conceptualized by Morgan [32] as a behavioral dysfunction and, therefore, as addiction that takes negative connotation. He assumed that excessive physical exercise could lead to physical damage and to neglect many everyday life contexts (e.g., family, job). There are two key-aspects of this condition: firstly, sport becomes a daily need; secondly, the presence of withdrawal symptoms in case of abstention from training.

Lukács et al. [33] explored exercise addiction in amateur runners through multidimensional approach, underlying some risk predictors of develop this addiction. The sample consisted in 257 runners with at least 2 consecutive years of practice. Risk prevalence of exercise addiction (EA) was 8.6%, while 53.6% of respondents was characterized as symptomatic non-addicted and 37.8% asymptomatic non-addicted. Likelihood ratio tests indicated that five factors have contributed in a significant way: time spent running weekly (*p* < 0.001); childhood activity *p* = 0.008); level of education (*p* = 0.006); anxiety (*p* = 0.023); loneliness (*p* = 0.004). It was observed that the risk group obtained a higher score in “lack of control” subscale; these runners were, in fact, less able to manage the urge of doing or to stop exercising. The results support the theory that assumes lonely athletes use sport activity as source of joy and happiness—to cope for anxiety and loneliness—increasing time or quantity of physical activities, because they need more and more of it to achieve these emotions.

A new and interesting discovery is that a lower level of education may predict the probability of exercise addiction. Studying at better universities or colleges may improve the capability to deal with emotional distress and develop coping strategies which, in their turn, can prevent behavioral disorders and presumably numerous other problems. In fact, the level of education seemed to be a protective factor, as resulted in Menczel’s research [34]: 65% of the sample—1743 subjects, 58.6% of which were female, the mean age was 31.7 (SD = 8.491), the youngest person was 18, and the oldest one was 61-year-old—had a university or college degree. The subjects were administered questionnaires consisted of different parts, namely, demographic questions, e.g., age, gender, residency, weight and height. In the second part, sporting habits were assessed, such as the frequency, the kind of sport they practiced. Menczel also measured the existence of eating disorders. As the final part of the survey, fitness users were asked to fill in two standardized questionnaires, the Exercise Addiction Inventory (EAI) and the Exercise Dependence Scale-21 (EDS). Additionally, to these scales—self-esteem, well-being and sensation seeking were also measured. Furthermore, body dissatisfaction was measured with the Eating Disorder Inventory and the SCOFF scale. In terms of education, the higher level of studies, the lower scores people obtained on exercise dependence (ED) (rs, 0.094, *p* ≤ 0.001; rs, 0.148, *p* ≤ 0.001). As Menczel suggests, addictive exercising may link to having worse coping mechanisms, poorer ways to deal with stress. One way to improve in it is to study in higher education.

#### 3.3.1. Exercise Addiction, Behavioral Disorders and Psychological Distress

Exercise addiction can also be related to certain personality characteristics and/or psychological distress. In a cross-sectional survey by Guidi et al. [35], a total of 79 participants (recruited in five gyms) completed the following self-report questionnaires: Exercise Dependence Questionnaire (EDQ), Eating Disorder Inventory II (EDI-2), Temperament and Character Inventory (TCI), Attitude Toward Self Scale (ATS), Muscle Dysmorphia Questionnaire (MDQ) and Symptom Questionnaire (SQ). In the sample, exercise addicted subjects were 32, who were compared with control subjects (n = 47). From the results, it was observed significant differences between genres in EDI-2 total score, where women have obtained higher scores than men (*p* = 0.048). Participants with primary exercise addiction showed more dysfunctional eating patterns than control group; in fact, significant differences emerged in EDI-2 total score (*p* < 0.001). Other differences between these groups are associated to behavioral aspects: participants with primary exercise addiction reached higher scores than control group in these TCI subscales: damage avoidance (*p* = 0.038), persistence (*p* = 0.024), self-directivity (*p* = 0.002). In contrast, lower scores were reached in matureness character index (*p* = 0.033). In SQ total score (*p* = 0.002) and in anxiety (*p* = 0.001) and hostility subscales (*p* < 0.001), better scores were found in participants with primary exercise addiction. Considering the issue of body dysmorphia related to exercise addiction, significant differences in ATS dysmorphophobia subscale (*p* = 0.010) emerged, with higher scores in participants with primary exercise addiction. Primary exercise addiction resulted significantly associated with higher scores in muscular dysmorphia, evaluated by MDQ (*p* < 0.001). Data provided further support to the idea that exercise addiction could be a specific clinical condition associated with psychological symptoms and personality characteristics. These evidences report a relation between excessive physical activity and eating behavioral disorders. Regarding personality characteristics, these results are consistent with those of other studies, in which a negative association between self-esteem and excessive physical activity was highlighted. The results also indicate difficulty in assumption of responsibility and lack of objectives. Finally, the presence of primary exercise addiction is associated with significant higher scores in muscular and body dysmorphia, anxiety and hostility.

In Hausenblas e Giacobbi’s hypothesis [22], some people could start to perform physical activity as a coping strategy towards psychological distress. The researchers therefore examined the relationship between personality and exercise dependence symptoms; participants of the study were 390 university students who completed multidimensional assessments of personality, exercise dependence, and exercise behavior. To examine the predictive relationship of personality for exercise dependence symptoms hierarchical regressions with forced block entry were undertaken. In Block 1, exercise dependence was regressed on exercise behavior. In Block 2, the personality subscales (neuroticism, extraversion, conscientiousness, agreeableness, openness) were entered into the regression. Results showed that extraversion, neuroticism, and agreeableness predicted exercise dependence symptoms.

#### 3.3.2. Exercise Addiction during COVID-19 Pandemic

The current COVID-19 pandemic has changed many aspects of everyday life. We have compared the few studies in literature which investigated the impact of the pandemic on exercise addiction and possible associated disorders. As a result, two studies—centered on predictors—are presented.

In a study conducted by Scharmer et al. [36], psychological consequences due to current pandemic situation were explored, intended as predictors to develop compulsive practices (e.g., eating disorders). Particularly, the objective of this study was to explore the association among anxiety caused by COVID-19, trait intolerance of uncertainty, COVID-19 intolerance of uncertainty, eating disorder and exercise addiction. Participants were 295 university students (M:19.7 years; SD: 2.0). The following tools were administrated: EDE—Q (Eating Disorder Examination Questionnaire); STAI—T (State—Trait Anxiety Inventory—Trait subscale); CET (Compulsive Exercise Test); IUS—12 (COVID-19 intolerance—uncertainty); GLETQ (Gordon Leisure—Time Exercise Questionnaire). CET high scores emerged and positively correlated only with the trait intolerance of uncertainty. People with lower traits and intolerance of uncertainty showed a strong association between COVID-19 anxiety and CET and EDE-Q scores. In fact, this study shows that trait intolerance of uncertainty is the strongest predictor of compulsive exercise, while anxiety trait is the strongest predictor of eating disorder.

In an experimental study [37], possible consequences that physical activity restraint during current COVID-19 pandemic can have on people with exercise addiction were investigated. The study was conducted using a sample of 1079 participants from eight Spanish language countries. To test the relation between risk of EA and weekly exercise variation, a bivariate correlation was calculated, which was weak but statistically significant (r = −0.127, *p* < 0.001), showing the existence of a weak correlation between the decrease of exercise during COVID-19 pandemic and risk of exercise addiction only if there was not control on passion and perfectionism factors; otherwise, the relation was not significant (r = −0.024, *p* > 0.05). The negative relation between risk of exercise addiction and variation of exercise during COVID-19 pandemic was supported only until passion and perfectionism were not under control. Eventually, a positive and statistically significant correlation was found between exercise addiction and passion and perfectionism (Table 2).

## 4. Discussion and Conclusions

Regarding the benefits of physical exercise on cognitive functions, we can say that most of the considered sample have greater precision and speed of response in information processing tasks; children have an improvement in executive functions, selective attention, linguistic understanding, and a wider lexical network, syntactic and spelling skills, working memory, cognitive flexibility, inhibition control and school performance.

Furthermore, a particular interest of our review was to explore the variable of the presence of ADHD in a sample of children, in which we found that short-term aerobic exercise, based on different formats of aerobic intervention, appears to be effective in mitigating symptoms such as attention, hyperactivity, impulsivity, anxiety, and may induce an improvement in executive functions and behavioral disorders at the socio-relational level.

Regarding another part of the sample, adults over 50 years old, physical exercise is inversely correlated to the risk of cognitive decline and the onset of dementia, while it would also favor a notable improvement in executive functions, visuospatial functions, episodic memory, fluidity of words, processing speed and global cognitive functions.

Finally, we investigated the importance of exercise in patients with schizophrenic spectrum disorder or bipolar disorder, where it was found that exercise dosage appears to be an important factor in achieving cognitive improvement, as previous studies demonstrated that the amount of exercise achieved by participants during an intervention is a significant predictor of cognitive improvements in overall functions, general intellectual skills, verbal memory, and working memory.

The second purpose of our review concerns exercise addiction and any individual and situational factors that have been considered as possible predictors for the onset of a sport addiction. Results showed that what may predict the occurrence of physical activity addiction may be high weekly time spent running, physical activity occurring during childhood, low education, high levels of anxiety and hostility, loneliness, low self-esteem, and gender (since we found greater incidence in the female gender). In addition, high levels of character aspects such as persistence and tenacity, a self-directed personality, avoidance of harm and intolerance of uncertainties.

Other risk elements for the onset of sport addiction are having developed dysfunctional eating patterns or eating disorders or expressing concerns resulting from body and muscle dysmorphisms.

A further prodrome is the impossibility of training, a factor contextualized in the current historical period, the advent of the COVID-19 Pandemic, even more so if associated with high levels of passion and perfectionism.

It is important to highlight the absence of specific studies concerning the cognitive consequences of exercise addiction. In fact, most of the literature currently available focuses on risk factors and other consequences, such as physical damage because of excessive and prolonged exertion and worsening of interpersonal or work relationships. We hypothesize that, since exercise addiction is considered a behavioral addiction, it may produce the following cognitive effects: constructs such as impulsivity, compulsivity, and attention regulation, which may also be relevant, applicable, and successful for understanding and subsequent treatment of behavioral addictions [38]. This, however, is only a hypothesis that should be explored in the future, and which can be a starting point for new research.

This review has some limitations to consider. First of all, presenting both healthy populations and clinical populations without any distinction may be a problem. In fact, in cognitive enhancement literature, it is sometimes shown that people with more performing cognitive functions at baseline show reduced enhancement as opposed to clinical populations. Besides, mechanisms of actions are not the same necessarily. For example, the neuroplasticity promoted by neurostimulation techniques among clinical populations can impact to a sufficient level to induce changes in behavior. However, among healthy populations with optimal homeostatic cortical activity, long-term alteration might be necessary to observe the effects of neuroplasticity on behavior [39]. Nevertheless, there are studies that claim the opposite, showing higher cognitive changes in non-clinical populations rather than in clinical populations at baseline [40]. This, therefore, requires further investigation in the future. Secondarily, we do not discuss the cognitive factors of sports addiction and about the risks ensuing from sports addiction. Eventually, a final limitation concerns the fact that the analysis of risk factors for developing exercise addiction during the COVID-19 pandemic refers to an event and a period still in progress, although, at the time of writing, lockdowns are no longer in effect.

In conclusion, these findings could lead to the dissemination of more informed policies on the use of PA to improve and shape cognitive functions throughout an individual’s life. In accordance with the compared literature, a more than beneficial effect of physical activity can be affirmed in promoting a condition of mental health, an enhancement of cognitive function in healthy individuals but also in a preventive and curative key to the well-being of those who are affected by certain psychic disorders. In addition, about the other side of the review, the importance of an adequate psychological-clinical evaluation of psychological distress and personality characteristics associated with exercise addiction stands out. Therefore, the right balance of sports practice could thus become a real coping strategy useful for facing the problems of daily life and for reducing stress and negative emotions, but in a functional way to the psycho-physical health of the subject.

## Figures and Tables

**Table 1:** Positive effects of physical activity on cognitive functions.

PARTICIPANTS	BENEFITS OF PHYSICAL ACTIVITY ON COGNITIVE FUNCTIONS
GENERAL POPULATION	Better precision and response speed in information processing tasks
CHILDREN	Improvement of executive functions Improvement selective attention Wider lexical network Improvement of linguistic understanding Improvement of syntactic ability Improvement of spelling skills Improved working memory Improvement of cognitive flexibility Improved inhibition control Improvement of school performance
CHILDREN WITH ADHD	Improvement of attention Less hyperactivity Less impulsiveness Improvement of executive functions
ADULTS OVER 50 YEARS OLD	Lower risk of cognitive decline and dementia Improvement of executive functions Improvement of visual-spatial functions Improvement of episodic memory Improved fluency of words Improvement of processing speed Improvement of global cognitive functions
PATIENTS WITH SCHIZOPHRENIC SPECTRUM DISORDER OR BIPOLAR DISORDER	Improvement of overall cognitive functioning Improvement of general intellectual skills Improvement of verbal memory Improved working memory

**Table 2:** Individual and situational factors and possible predictors of exercise addiction development.

EXERCISE ADDICTION: INDIVIDUAL AND SITUATIONAL FACTORS AND POSSIBLE PREDICTORS
High time spent exercising weekly
Physical activity occurred in childhood
Low level of education
High levels of anxiety
High levels of hostility
Loneliness
Low self-esteem
Gender (higher incidence among women)
High levels of character aspects such as: persistence, avoidance of harm, self-direction and intolerance of uncertainty
Prolonged periods of inability to train (for example, COVID-19 pandemic) when associated with high levels of passion and perfectionism
Dysfunctional eating patterns and eating disorders
Body and muscle dysmorphisms

## Data Availability

Not applicable.
